# Enhancing the Nutritional Value and Antioxidant Activity of *Auricularia polytricha* Through Efficient Utilization of Agricultural Waste

**DOI:** 10.1155/ijfo/7257263

**Published:** 2025-12-09

**Authors:** Zaili Qin, Nan Wu, Entaj Tarafder, Shihui Mei, Jiangtao Xie, Changtian Li, Fenghua Tian

**Affiliations:** ^1^ Department of Plant Pathology, College of Agriculture, Guizhou University, Guiyang, China, gzu.edu.cn; ^2^ Institute of Edible Mushroom, Guizhou University, Guiyang, Guizhou, China, gzu.edu.cn; ^3^ School of Life Science and Biotechnology, Heilongjiang Bayi Agricultural University, Harbin, China, hlau.cn; ^4^ Engineering Research Center of the Chinese Ministry of Education for Edible and Medicinal Mushroom, Jilin Agricultural University, Changchun, China, jlau.edu.cn

**Keywords:** agricultural waste utilization, *Auricularia polytricha*, edible mushroom, nutritional composition, straw degradation technology, wood-rotting

## Abstract

*Auricularia polytricha*, a nutritious edible wood‐rotting mushroom, faces cultivation challenges due to the limited availability of wood chips. It is urgent to find suitable flat substitutes to replace the current material. This study explores the use of 12 types of agricultural waste as alternative growth substrates, analyzing their effects on the physiological and biochemical characteristics of both mycelia and fruiting bodies. The agricultural waste that demonstrated greater suitability for the growth of *A. polytricha* was then selected as a substrate to evaluate its effect on the nutritional composition and antioxidant capacity of the fruiting bodies. The research findings have highlighted the potential for cotton straw, coix seed straw, and wheat straw to serve as the most efficient substrates in the cultivation of *A. polytricha*. The utilization of agricultural waste as a growth medium has been found to markedly enhance the activity of enzymes such as laccase, cellulase, and polyphenol oxidase within the mycelia, resulting in a significant reduction of the cultivation cycle by 16 days. These substrates also improved the nutritional composition of fruiting bodies, increasing crude fat, crude protein, total sugars, and mineral contents of iron (Fe) and zinc (Zn) in the fruiting bodies, with increases of 1.6‐fold, 2.6‐fold, 2.2‐fold, fourfold, and sevenfold, respectively. Additionally, the in vitro antioxidant activity of *A. polytricha* was assessed, revealing an enhancement in the DPPH free radical scavenging ability by up to 36.06%. This study highlights the utilization of agricultural waste to enhance the nutrient profile of *A*. *polytricha*, providing innovative approaches for optimizing its production. Additionally, it offers significant insights into advancing technologies related to “transforming wood‐rotting mushrooms into agents for straw degradation.”

## 1. Introduction

*Auricularia polytricha*, commonly recognized as an edible mushroom, is celebrated for its gelatinous yet meaty texture. It has earned the moniker “meat of the vegetarian” due to its satisfyingly crunchy and resilient bite, coupled with its rich nutritional profile [1, 2]. In 2022, China produced 1.8173 million t of *A*. *polytricha*, making it the seventh most cultivated edible mushroom in the country (data source: China Edible Mushroom Association). Research has highlighted its abundance in polysaccharides, which exhibit antioxidant, antilipid, antitumor, and antiaging properties [3]. Notably, polysaccharides AAPs‐1, AAPs‐2, and AAPs‐3 extracted from *A*. *polytricha* have shown inhibitory effects on S180 sarcoma in mice, with inhibition rates of 12.0%, 40.4%, and 26.2%, respectively [4, 5]. *A. polytricha* is a hardy species that flourishes in high‐temperature environments and exhibits remarkable resilience to cold temperatures, which simplifies its cultivation process. This adaptability has facilitated its widespread cultivation across various countries globally, with significant production hubs primarily in China, South Korea, and Japan [6]. With ongoing in‐depth research into its nutritional value, the cultivation scale of *A*. *polytricha* continues to expand rapidly. However, increased national efforts in ecological conservation have intensified the “mushroom–forest conflict,” particularly due to a traditional shortage of wood chips for cultivation. The rising production costs, driven by limited wood chip availability, pose significant challenges to the mushroom industry. Addressing this issue by developing cost‐effective cultivation materials has become a pressing necessity.

The straw mushroom industry encompasses the cultivation of edible and medicinal fungi using agricultural crop residues as the primary substrate [7]. This sector significantly contributes to the sustainable utilization of agricultural byproducts, contributing to both the economy and the environment. Globally, around 6 billion t of agricultural waste are generated annually, with China contributing nearly 1 billion t. This waste primarily consists of residues from grain crops and cash crops [8]. The vast straw resources are predominantly used in five key ways: as fertilizer, fodder, fuel, substrate, and raw material. However, using straw as fertilizer and fuel can increase greenhouse gas emissions. Additionally, its use as fodder is limited due to high lignification, making it less palatable and harder for livestock to digest [9–12]. The mushroom cultivation industry relies on a variety of industrial and agricultural byproducts, including wood chips, fruit tree prunings, and crop residues like cotton waste. These substrates are rich in lignin, cellulose, and hemicellulose, as well as essential nutrients such as phosphorus (P) and potassium (K). Not only do these constituents serve as essential nutrients for crop health, but they also play a foundational role in fueling microbial proliferation and the fermentation dynamics [13, 14, 16]. Agricultural byproducts, including corn and wheat straw, are abundant in cellulose and lignin, which are particularly well suited for mushroom cultivation. Mushrooms act as critical decomposers in ecosystems, transforming agricultural waste into edible food products. This not only supports natural ecological cycles but also offers an eco‐friendly and efficient solution for managing agricultural waste. Moreover, the industry bridges the gap between sustainable agriculture and waste management, promoting environmental and economic benefits.

Currently, agricultural waste straw, such as corn straw, rice straw, wheat straw, sugar cane straw, rape straw, and soybean straw, is widely used in edible mushroom cultivation, with corn, rice, and wheat straw comprising over 75% of total straw resources [15]. Different edible mushroom species have specific substrate preferences and unique growth characteristics. For example, cotton straw is particularly suitable for cultivating *Agrocybe cylindracea* and *Pleurotus ostreatus* [17], while wheat straw, bagasse, and sawdust are ideal for the growth of *Hericium erinaceus* [18]. Additionally, using distillery and olive mill waste as substrates can enhance the production of secondary metabolites, such as triterpenes and amino acids, in *P*. *citsrinopileatus*, improving its antioxidant activity [19]. The composition of the substrate significantly impacts the growth of edible mushrooms. D′Agostini et al. [20] demonstrated that a carbon‐to‐nitrogen (C/N) ratio below 30% results in the inhibition of mycelial growth due to tamarind laccase activity. The use of agricultural waste not only supports sustainable agricultural practices and reduces pollution but also provides valuable insights for cultivating functional edible mushrooms. Such practices are critical for diversifying mushroom cultivation and enhancing their nutritional and economic value.

The objective of this research was to examine agricultural wastes suitable for cultivating *A. polytricha* and to optimize substrate composition for its growth. The study is aimed at investigating how different cultivation formulations influence extracellular enzyme activities, agronomic characteristics, nutritional value, and antioxidant properties of *A. polytricha*. This initiative not only offers an eco‐friendly approach to recycling agricultural waste straw, thereby significantly reducing environmental pollution caused by agricultural waste, but also lays a theoretical foundation for developing a high‐quality, cost‐effective, nutrient‐rich, and multifunctional production system for *A. polytricha*.

## 2. Materials and Methods

### 2.1. Fungal Culture and Chemical Reagents

In this study, a commercial strain of *A. polytricha*, designated as GUCCTM142, was obtained and authenticated by the College of Agriculture at Guizhou University, China. This strain is preserved in potato dextrose agar (PDA) at the Culture Collection of the Department of Plant Pathology, Agriculture College, Guizhou University (GUCC). For the subsequent experimental procedures, the fungal cultures were revitalized from the stored PDA cultures and incubated at 24°C.

To identify suitable crop residues for cultivating *A. polytricha*, the research team acquired a range of materials, including soybean straw, coix seed straw, sorghum straw, wheat straw, corn straw, rice straw, rice husk, bagasse, peanut straw, cottonseed hull, cotton straw, corn cob, and hardwood chips. Additionally, the study necessitated the use of various chemicals for evaluating physiological markers, such as ferrous sulfate, K persulfate, vitamin C, salicylic acid, K ferricyanide, ferric chloride, and other reagents. All these chemicals were obtained from Xingyao Biotechnology Company, Guiyang, China.

### 2.2. Screening of Straw Substrates for *A. polytricha* Cultivation

To assess the impact of various straw substrates on the mycelial growth of *A. polytricha*, precisely 30 g of each straw powder type—soybean, coix seed, sorghum, wheat, corn, rice, rice husk, bagasse, peanut, cottonseed hull, cotton, and corncob—was measured. These were used to substitute the sawdust in the foundational formula, which consists of coarse sawdust (78%), wheat bran (20%), lime (1%), and gypsum (1%). Hardwood served as the control substrate. The moisture level was standardized at 60%, and the mixtures were uniformly distributed into glass dishes with a 10 cm diameter. Each type of straw medium was prepared in quintuplicate, sterilized in an autoclave at 121°C for 1 h, and inoculated once they reached room temperature. [21, 22].

Utilizing rigorous aseptic methods, a 5‐mm‐diameter hole punch was meticulously used to craft evenly distributed holes along the edge of the PDA plates colonized by the mycelium. The mycelial plugs, thus harvested, were subsequently transferred into the sterilized substrate mediums. The growth of the mycelium was closely monitored, and the extent of its proliferation was measured on the 12th day following inoculation.

### 2.3. Fungal Strain Cultivation and Mushroom Cultivation Protocol

The liquid medium was composed of a nutrient blend with the following concentrations: potato at 100 g/L, wheat bran at 40 g/L, glucose at 20 g/L, peptone at 2 g/L, brown sugar at 15 g/L, MgSO_4_ at 1 g/L, and KH_2_PO_4_ at 2 g/L. Activated mycelia of 5 mm diameter were harvested for the inoculation of liquid culture strains. For the preparation of liquid spawn, mycelial plugs, each measuring 5 mm in diameter, were placed at a rate of 6 per flask into 1‐L flasks prefilled with 400 mL of the prepared liquid medium. These flasks were then incubated in a shaker (Bluepard, Shanghai, China) operating at a speed of 160 rpm at a constant temperature of 24°C in complete darkness for a period of 8 days.

Formulation design was as follows: The simplex‐lattice design was used to design the upper and lower bounds of the limiting components in the Mixture design. The software Design‐Expert 8.0.6.1 was used to optimize the proportion of each component in the main ingredient. *X*_1_–*X*_3_ were set at 0%–100%, *X* was the main ingredient, the main ingredient accounted for 78% of the entire medium, and the remaining components, including wheat bran, lime powder, and gypsum powder, made up 22% of the total mixture. *X*_1_ was cotton straw, *X*_2_ was coix seed straw, and *X*_3_ was wheat straw, *X*_1_ + *X*_2_ + *X*_3_ = 100*%*. The control formula was as follows: coarse sawdust 78%, wheat bran 20%, lime 1%, and gypsum 1% (Table 1).

**Table 1:** Formulation design by the simple lattice method.

**Substrate no.**	**C/N**	**X**_1_	**X**_2_	**X**_3_
T1	31	100	0	0
T2	55	0	0	100
T3	46	0	100	0
T4	42	50	0	50
T5	38	50	50	0
T6	49	0	50	50
T7	44	33.33	33.33	33.33
T8	45	16.67	66.67	16.67
T9	50	16.67	16.67	66.67
T10	38	66.67	16.67	16.67
T11	39	51.97	7	41.03
T12	52	8.97	83.33	7.7

According to the base mass required for each formula, the samples were weighed and repeatedly stirred and mixed, and then, water was added until the water content was 60%. The samples were divided into polypropylene bags (158 × 370 × 32 mm, 0.5 kg/bag). The samples were sterilized in a 121°C pressure cooker (LGB‐1500*4500, Lianggong, Zhucheng, China) for 3 h. After the bacterial package was cooled to room temperature, the liquid strain was inoculated at 15 mL/bag, and the sponge was sealed for dark culture.

Mycelial culture was as follows: Cultivation is performed in the absence of light, beginning with a temperature range of 25°C–28°C, which is subsequently lowered to 20°C–25°C for the later stages. The relative humidity is consistently maintained between 50% and 60% throughout the entire cultivation period. Postripening and piercing were as follows: Following a 10‐day postripening period that allows for complete mycelial development, the cultivation bags are punctured with an average of 90–100 holes per bag, each hole reaching a depth of 0.5 cm. A stationary rod is introduced to minimize disturbance, and watering is avoided during the initial week. The environmental conditions are meticulously controlled to ensure a warm and humid atmosphere, promoting mycelial recovery and the formation of fruiting body primordia. The primordial differentiation phase was as follows: This phase takes place 7–10 days after piercing, with a focus on inducing the base of the fruiting body. The relative humidity of the air above the substrate is carefully sustained at around 80%, with temperature management between 20°C and 25°C. The conditions are optimized with the provision of diffused light and ample ventilation to support primordium formation. The fruiting body growth phase was as follows: Commencing from the initial appearance to the full development of the fruiting bodies, this stage involves a gradual increase in water supply. The air′s relative humidity is raised to 90%–100%, and ventilation is enhanced to support the growth of the fruiting bodies. Harvesting is carried out once the fruiting bodies have reached maturity.

### 2.4. Assessment of Extracellular Enzyme Activity

To ascertain the capacity of *A. polytricha* mycelia to metabolize various substrate matrices, the activities of key extracellular enzymes—laccase, polyphenol oxidase, and cellulase—were measured during the mycelial, ripening, and primordium stages of cultivation for each substrate formula. The procedure for extracting crude enzyme solution was as follows: 2 g of fungal substance was accurately weighed and then transferred to a 50‐mL centrifuge tube. Subsequently, 20 mL of extraction buffer was added. The mixture was incubated in a water bath maintained at 37°C for 2 h to allow for enzyme extraction. After incubation, the mixture was filtered, and the filtrate′s volume was adjusted to 25 mL. The solution was then centrifuged at 4°C at a speed of 8000 rpm for 10 min to pellet the cellular debris. The resulting supernatant, rich in extracellular enzymes, was collected as the crude enzyme solution for further analysis [21, 22]. This method ensures the isolation of extracellular enzymes, providing a basis for evaluating the enzymatic activity related to substrate degradation and utilization by the fungal mycelia.

#### 2.4.1. Detection of Laccase Activity

Laccase activity was determined using a standardized assay where the quantity of enzyme that oxidizes 1 μmol of ABTS per minute per gram of the sample is defined as one unit of laccase activity, denoted as *U*. To conduct the assay, 0.2 mL of the previously extracted crude enzyme solution is aliquoted, to which 0.7 mL of distilled water, 0.45 mL of sodium acetate buffer (0.1 mol/L at pH 5.0), and 0.15 mL of ABTS solution (1 mmol/L) are added. The mixture is then quickly brought to a 30°C water bath for incubation at this temperature for 3 min. The reaction is monitored spectrophotometrically at 420 nm to quantify the laccase activity [23].

#### 2.4.2. Detection of Polyphenol Oxidase Activity

Polyphenol oxidase activity was quantified as follows: Each gram of sample absorbance value change per minute was 0.001 for 1 polyphenol oxidase viability unit, indicated in *U*. Take 1 mL of crude enzyme solution, add 5 mL of phosphate buffer (0.2 mol/L, pH 6.0) and 2 mL of catechol solution (0.12 mol/L), quickly put into a cold water in 37°C bath for 15 min, stand for 3 min to stop the reaction, and determine the absorbance value at 410 nm [24].

#### 2.4.3. Cellulase Activity Detection

Cellulase activity was measured using 15 mg/mL of reference sodium carboxymethyl cellulose and sodium acetate buffer (0.1 mol/L, pH 5.5) as substrate. Take 1 mL to be tested in a 50‐mL centrifuge tube, add 2 mL sodium carboxymethyl cellulose, put into 37°C water bath for 30 min, add 5 mL DNS reagent, vortex for 3 s, put into boiling water bath for 5 min, add 25 mL, determine the absorbance, and substitute the absorbance value into the standard curve to calculate the machine concentration C. Cellulase activity was expressed in gram (U/g) and was defined as the amount of enzyme required to release 1 *μ*mol of reducing sugar during minute of sodium carboxymethyl cellulose solution [25].

### 2.5. Evaluation of Agronomic Traits

The effects of different proportions of substrates on the growth cycle of *A. polytricha* were evaluated by mixing suitable straw with different proportions. This study mainly evaluated the following parameters [21, 22, 26]:

Harvest time day=fruiting body harvest date−bacterial inoculation date,Single package yield g=the weight of fresh fruiting body pieces produced by the fungus package,

### 2.6. Determination of Nutritional Components

The collected crop straw was dried at 50°C in the oven and then crushed through a 40‐mesh sieve. The carbon content in straw was determined by the sulfuric acid–K dichromate oxidation method. The nitrogen content in sawdust was determined by the Kjeldahl method.

Before chemical analysis, unless otherwise stated, the fruiting body samples of *A. polytricha* were dried to a constant weight at 60°C for 24 h and then converted into powder. The crude protein content was determined by the Kjeldahl method according to the Chinese food standard GB5009.5‐2016, and the crude fat content was determined by the Soxhlet extraction method according to the Chinese food standard GB/T 15674‐2009. The polysaccharide content was determined according to the method of Song et al. [27]. According to the method of the agricultural industry standard NY/T 1653‐2008 of the People′s Republic of China, the content of mineral elements was determined by inductively coupled plasma emission spectrometry (Shimadzu, Hong Kong, China).

### 2.7. Antioxidant Activity Assays

#### 2.7.1. ABTS Free Radical Scavenging Activity

The ABTS free radical scavenging ability was determined by mixing ABTS solution (7.4 mmol/L) with K persulfate (2.6 mmol/L) in equal volume and standing in the dark for 12–16 h. The ABTS working solution was prepared by diluting with 95% ethanol (about 1:30), and the absorbance at 734 nm was 0.70 ± 0.02. The 160 *μ*L ABTS working solution was gently mixed with 40 *μ*L test samples of different concentrations (0.5–2.5 mg/mL) and reacted at room temperature in the dark for 6 min. The absorbance value was measured at 734 nm with a microplate reader (1530) at 200 *μ*L. The samples were replaced with ethanol as a blank control. The ABTS working solution was replaced by 95% ethanol as the control group [28]:

(1) ABTS clearance=1−A1−A2A0×100%,

where *A*_0_ was the absorbance of the blank group, *A*_1_ was the absorbance of the sample group, and *A*_2_ was the absorbance of the control group.

#### 2.7.2. Hydroxyl Radical (•OH) Scavenging Activity

The method for the determination of •OH scavenging rate [29] was to take 2 mL of different concentrations of test samples (0.5–2.5 mg/mL) in a centrifuge tube, add 2 mL of 9 mmol/L ferrous sulfate solution, 2 mL of 9 mmol/L salicylic acid–ethanol solution and 2 mL of 8.8 mmol/L hydrogen peroxide solution, shake them up, place them in a water bath at 37°C for 30 min, and take 200 *μ*L. The absorbance *A*_1_ was measured at 517 nm on a microplate reader (1530). The sample was replaced with distilled water, and the same reagent was added. The absorbance was measured and recorded as *A*_0_. Anhydrous ethanol was used instead of salicylic acid–ethanol solution, and the same reagent was added. The absorbance was measured and recorded as *A*_2_. The calculation formula of the •OH scavenging rate was as follows:

(2) Scavenging activity %=1−A1−A2A0×100%,

where *A*_0_ was the absorbance of the blank group, *A*_1_ was the absorbance of the sample group, and *A*_2_ was the absorbance of the control group.

#### 2.7.3. Reducing Power

The reducing power of *A. polytricha* fruiting body was to mix different concentrations of test samples (0.5–2.5 mg/mL) with 2 mol/L pH 6.6 phosphate buffer (1 mL) and K ferricyanide (1 mL, 1%), and then, the mixture was reacted at 50°C for 20 min. Then, 2.5 mL trichloroacetic acid (10%) was added to stop the reaction. At the same time, distilled water (2.5 mL) and FeCl_3_ (0.5 mL, 0.1%) were added. After mixing, the mixture was allowed to stand for 10 min at room temperature. Subsequently, the absorbance was measured at 700 nm in a microplate reader (1530). In addition, VC was used as a positive control [3].

(3) Total reducing power=A2−A1,

where *A*_1_ was the absorbance of the sample and VC and *A*_2_ was the absorbance value of distilled water instead of the sample.

### 2.8. Statistical Treatment of Experimental Data

Excel software was used for data statistics. All experiments were a completely randomized design and repeated more than three times. Analysis of variance (ANOVA) was used to evaluate the results, and Duncan′s test was used to determine the significant difference between the arithmetic means with a probability of 5%. The results were expressed as mean ± SD. Plot with GraphPad Prism 9.5.1.

## 3. Results and Discussion

### 3.1. Assessment of Cultivation Parameters

#### 3.1.1. Assessment of *A. polytricha* Utilization of Various Agricultural Wastes

Table 2 evaluates the agricultural straw utilization capacity of *A. polytricha*. Mycelial growth rate analysis across straw substrates revealed significantly faster colonization on cotton straw (2.62 ± 0.22 mm/day; *p* < 0.05) versus other materials. Subsequent effective substrates included coix seed straw (2.50 ± 0.23 mm/day) and wheat straw (2.39 ± 0.48 mm/day). Notably, mycelial margins exhibited uniform morphology across all straw‐based substrates. In edible mushroom cultivation, crop straws function as fast‐acting substrates, whereas wood chips serve as slow‐release matrices [30]. This demonstrates *A. polytricha*′s metabolic efficiency in utilizing rapidly available nutrients from straw substrates to accelerate mycelial expansion.

**Table 2:** Effect of different crop straw on mycelial growth of *A. polytricha.*

**Substrate**	**Mycelial growth rate (mm·day** ^**−1**^ **)**	**Mycelial growth vigor**	**Edge tidiness of the colony**
Cotton straw	2.62 ± 0.22^a^	Vigorous, thicker	+++++
Rice shell	0.89 ± 0.12^fg^	Vigorous, thicker	+++
Sorghum pole	1.86 ± 0.27^cd^	Vigorous, thicker	+++
Corn straw	2.18 ± 0.06^bc^	Vigorous, thicker	+++++
Soybean pole	2.31 ± 0.27^ab^	Vigorous, thicker	+++++
Rice pole	2.16 ± 0.32^bc^	Sparse	+++
Coix seed straw	2.5 ± 0.23^ab^	Vigorous, thicker	+++++
Bagasse	1.2 ± 0.04^ef^	Vigorous, thicker	+++++
Peanut shell	1.30 ± 0.10^e^	Vigorous, thicker	+++
Wheat straw	2.39 ± 0.48^ab^	Vigorous, thicker	+++++
Corncob	1.75 ± 0.24^d^	Vigorous, thicker	+++++
Cottonseed hulls	1.71 ± 0.18^d^	Vigorous, thicker	+++
Miscellaneous sawdust (CK)	0.59 ± 0.08^g^	Sparse	+

*Note:* Values are expressed as means ± standard deviation (*n* = 3). Lack of letters in common indicates statistically significant differences (Duncan′s test, *p* < 0.05) for comparison of treatment means between different substrates. +++++, high; +++, moderate; +, weak.

#### 3.1.2. Influence of Straw Composition Variation on *A. polytricha* Mycelial Growth

This study employed a simplex‐lattice design to formulate 10 straw substrate formulations (Table 1) for investigating their effects on *A. polytricha* mycelial growth. As shown in Figure 1a, mycelia successfully colonized all 10 substrates, though significant growth variations occurred among formulations (*p* < 0.05). Mycelial development depends fundamentally on substrate nutritional composition. Figure 1b reveals that all formulations except T2 exhibited significantly higher growth rates than the wood chip control (CK), indicating superior nutrient acquisition efficiency from straw substrates.

Figure 1Effect of straw substrate on mycelial growth of *A. polytricha*. (a) Mycelial growth status; (b) Analysis of significant difference in mycelial growth rate of 11 formulas. (c) The interaction of three main materials affects the contour map of mycelial growth rate ((A) cotton straw, (B) coix seed straw, and (C) wheat straw). (d) Effects of combinations of cotton straw and coix seed straw on mycelial growth. (e) Effects of combinations of coix seed straw and wheat straw on mycelial growth. (f) Effects of combinations of cotton straw and wheat straw on mycelial growth. (Vertical axes in (d–f) represent mycelial growth rates, with horizontal axes designating substrates: (A) cotton straw, (B) coix seed straw, and (C) wheat straw.)

**Figure:**
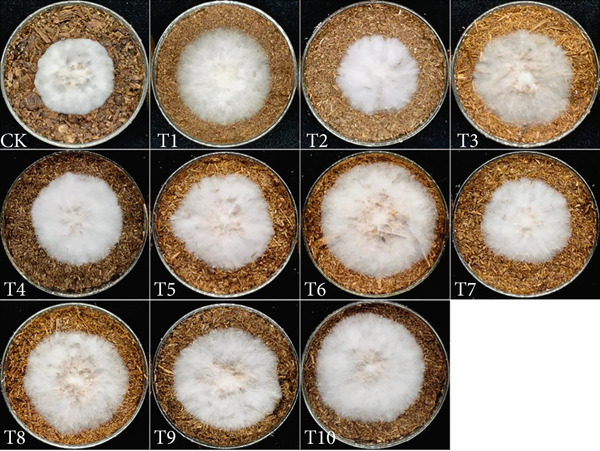
(a)

**Figure:**
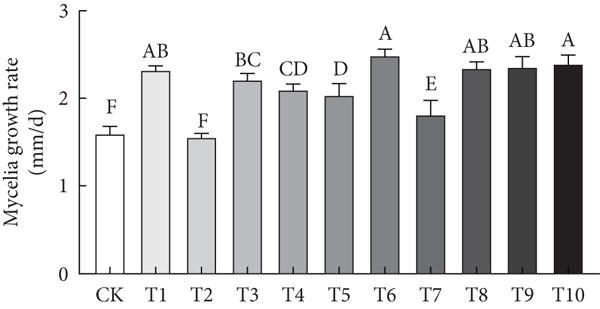
(b)

**Figure:**
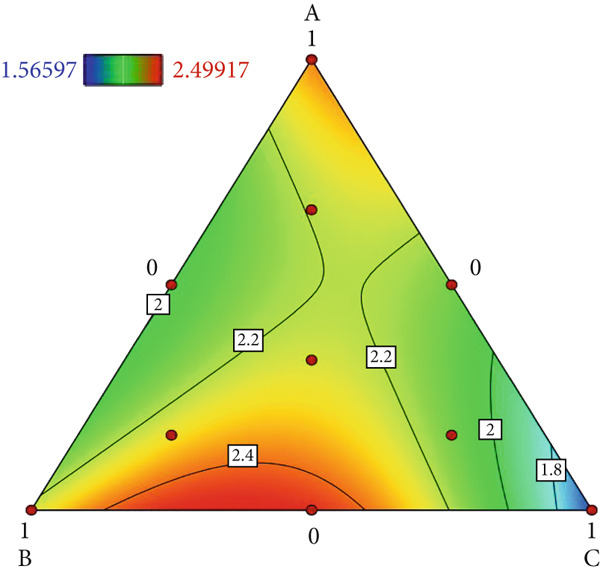
(c)

**Figure:**
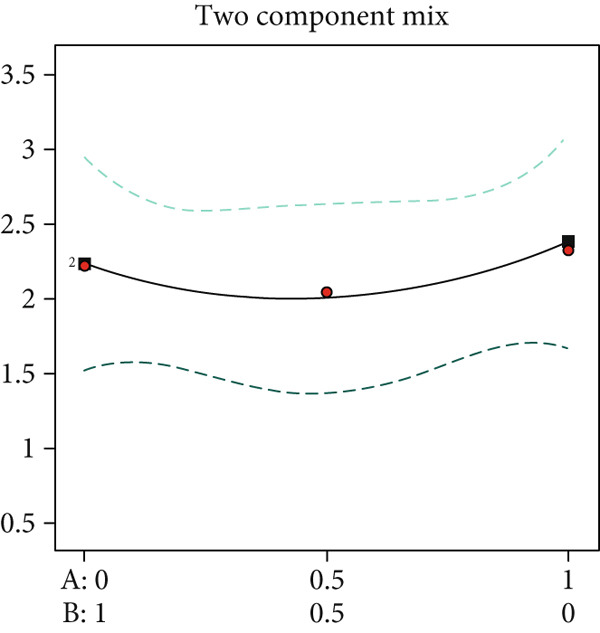
(d)

**Figure:**
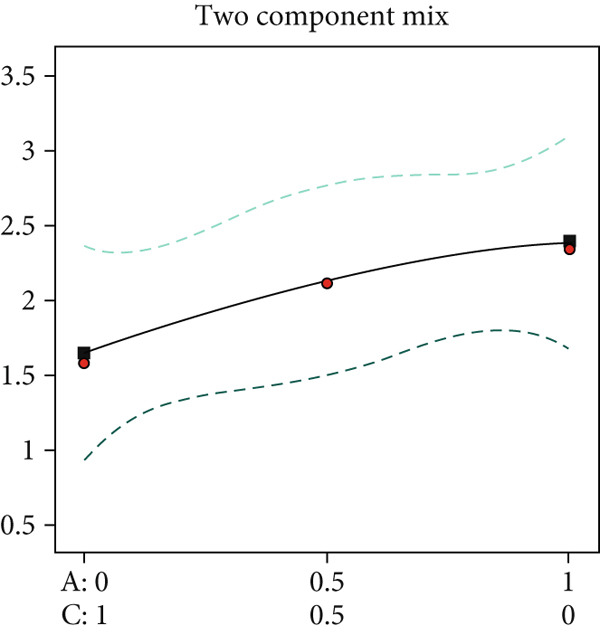
(e)

**Figure:**
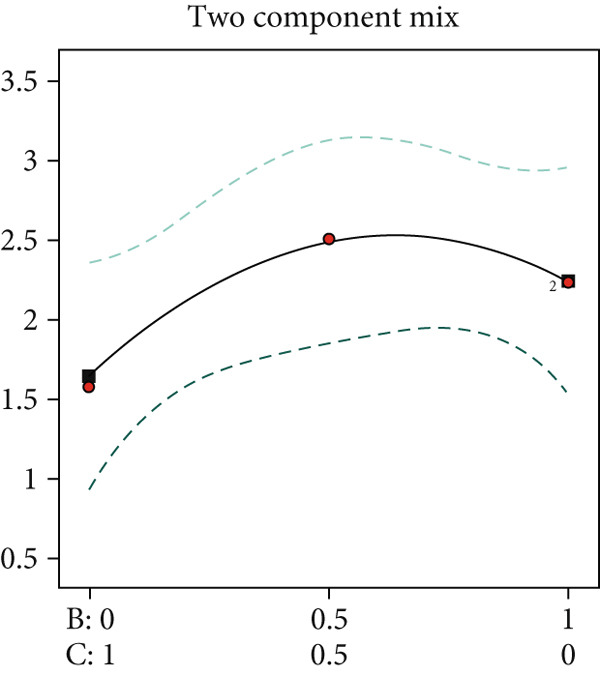
(f)

Substrate matrix interactions critically regulate mycelial growth [30]. Specifically, formulations predominantly composed of coix seed straw and wheat straw showed enhanced growth with increasing coix straw proportion. Conversely, pure wheat straw substrates demonstrated comparatively slower colonization versus mixed formulations (Figure 1c).

Edible mushroom mycelia exhibit substrate matrix selectivity during growth. When provided with nutritionally diverse substrates containing optimal nutrient ratios and concentrations, mycelia demonstrate accelerated and robust growth [15]. Our data reveal that combinations of cotton straw with coix seed straw (Figure 1d) or wheat straw (Figure 1e) were significantly less effective than the 1:1 coix seed straw:wheat straw formulation (Figure 1f). This indicates enhanced utilization efficiency of coix–wheat straw blends by *A. polytricha* mycelia.

Mixed straw substrates consistently outperform single‐substrate systems in promoting mycelial colonization. Xiao et al. [31] documented superior morphological characteristics (including improved coloration and delayed browning) in *Agaricus bisporus* fruiting bodies cultivated on rice–wheat straw mixtures versus wheat straw alone. Similarly, wheat–rapeseed straw combinations significantly enhance *P. djamor* mycelial growth rates [32].

#### 3.1.3. Influence of Agricultural Waste Cultivation on Extracellular Enzyme Activities

The effect of substrate composition on extracellular enzyme activities of *A. polytricha* is shown in Figure 2. In the culture systems with different substrate formulations, across cultivation systems with varied formulations, three key lignocellulose‐degrading enzymes exhibited distinct activity profiles: Laccase fluctuated between 0 and 150 U/g dry substrate (Figure 2a), cellulase was maintained at 0–20 U/g (Figure 2e), while polyphenol oxidase showed significantly higher activity (0–1000 U/g; Figure 2i). These differences in enzyme activities indicate that there is a significant differentiation in the lignocellulose‐degrading capacities of these three enzymes during the degradation of crop straw.

Figure 2Changes in the enzyme activity of *A. polytricha* in different straw matrix formulas. (a) Changes in laccase activity. (b–d) The Laccase mycelia stage, the postripening stage, and the primordium stage of the interaction of the main ingredients affect the laccase activity contour map. (e) Changes in cellulase activity. (f–h) The interaction of the main materials in the cellulase mycelia stage, the postripening stage, and the primordium stage affected the cellulase activity contour map. (i) Changes of polyphenol oxidase activity. (j–l) The interaction of the main materials in the polyphenol oxidase mycelia stage, the postripening stage, and the primordium stage affected the polyphenol oxidase activity contour map. (Ternary diagrams in (b–l) depict substrate composition effects, with apices *A* = *X*1, *B* = *X*2, and *C* = *X*3 representing cotton straw, coix seed straw, and wheat straw, respectively.)

**Figure:**
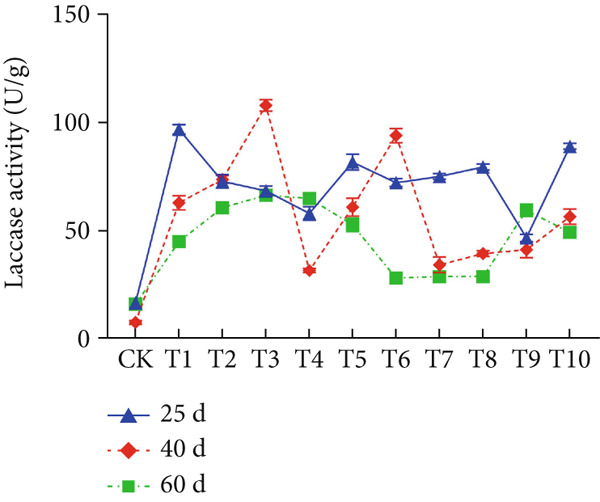
(a)

**Figure:**
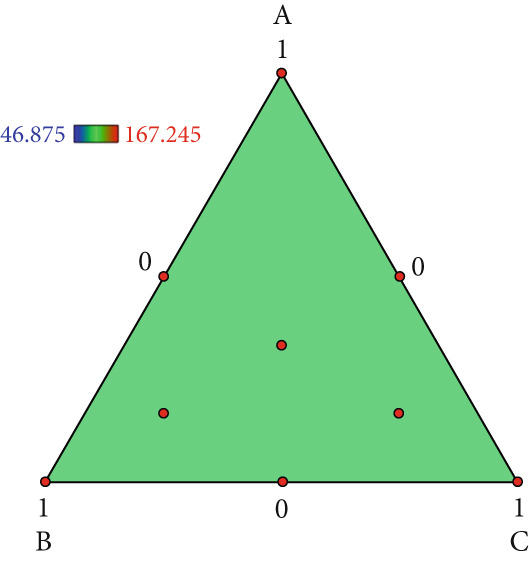
(b)

**Figure:**
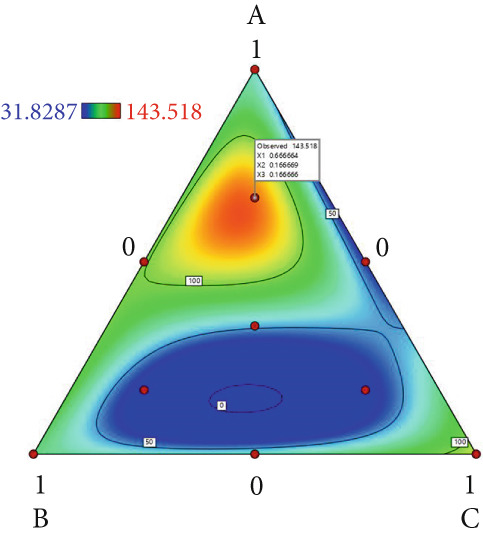
(c)

**Figure:**
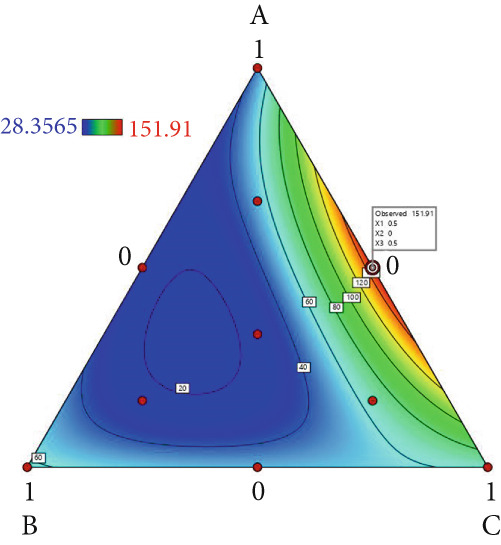
(d)

**Figure:**
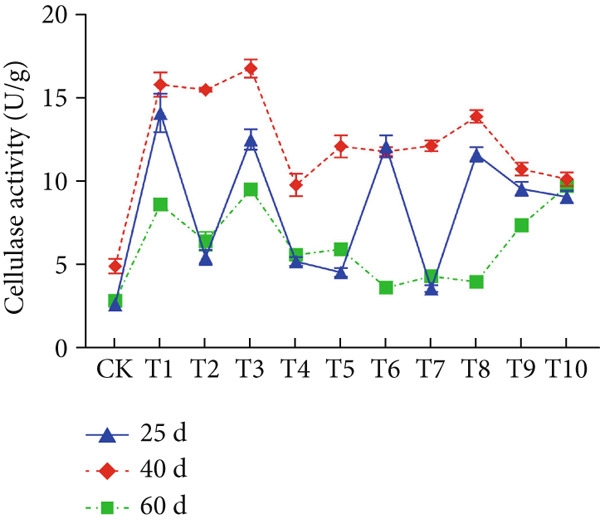
(e)

**Figure:**
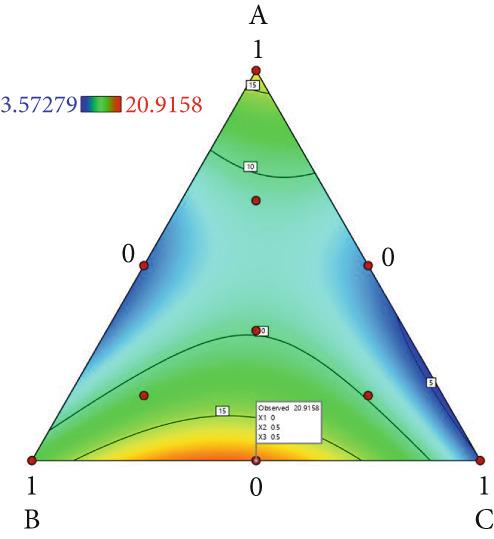
(f)

**Figure:**
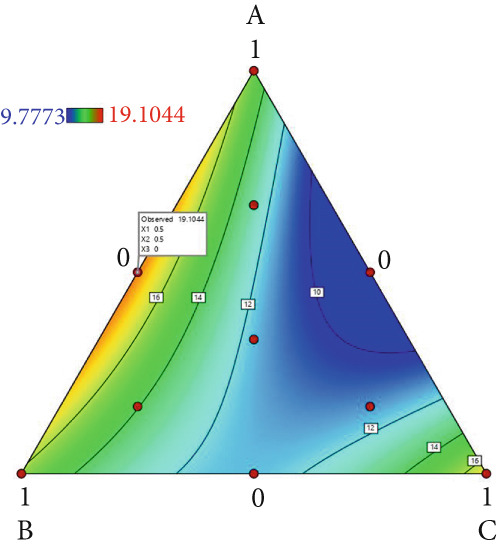
(g)

**Figure:**
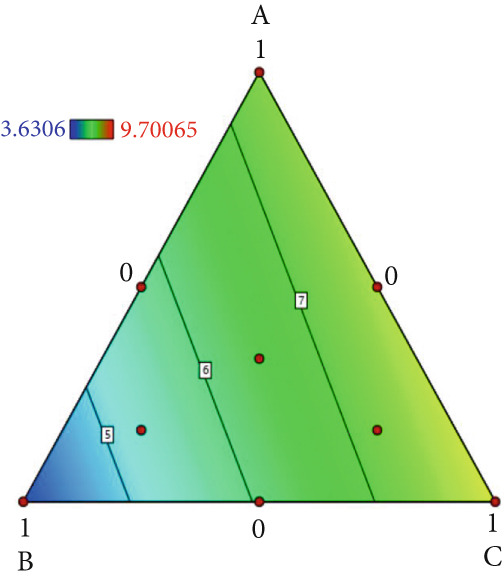
(h)

**Figure:**
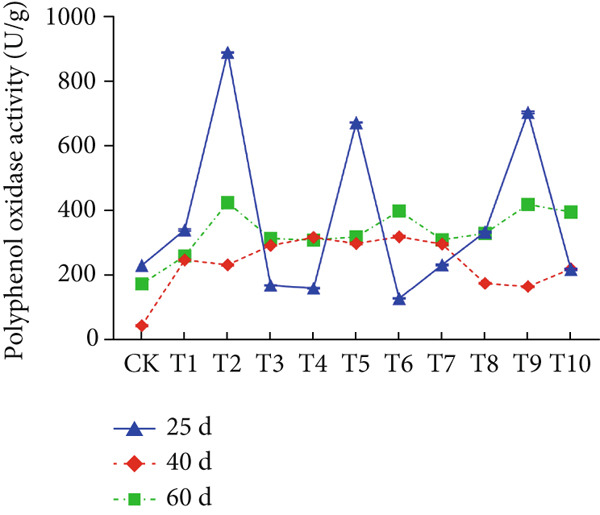
(i)

**Figure:**
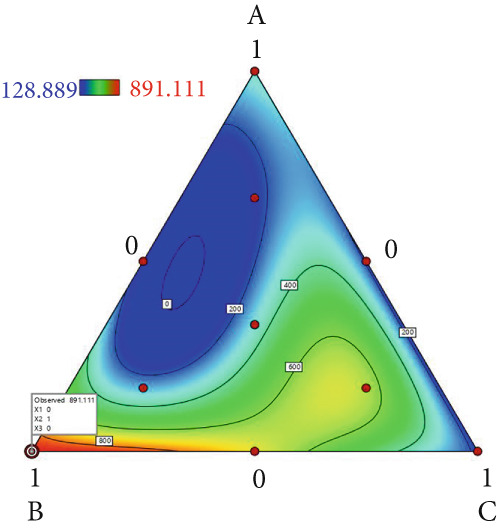
(j)

**Figure:**
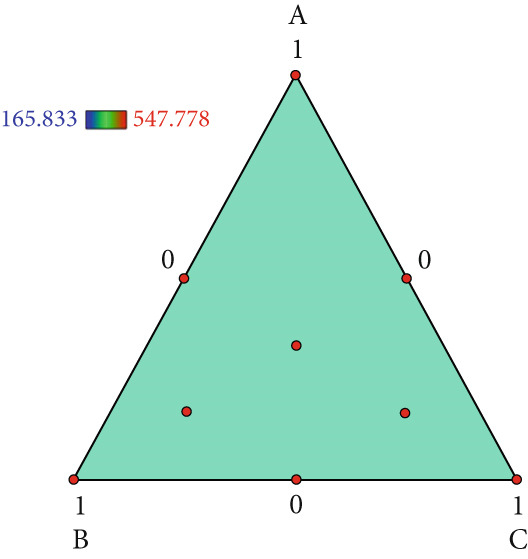
(k)

**Figure:**
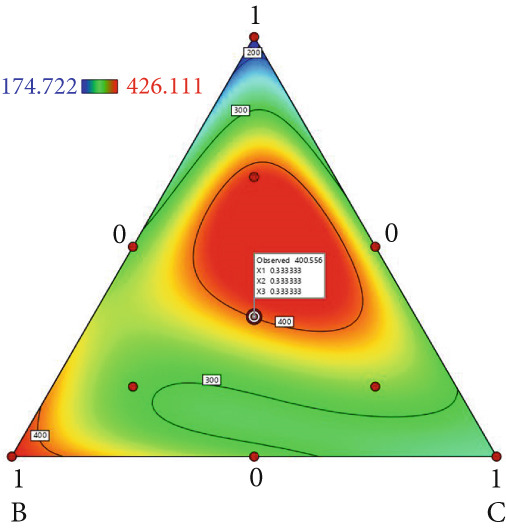
(l)

Lignocellulose constitutes the primary recalcitrant component in straw biomass, limiting its biodegradation efficiency [33]. To utilize this substrate, fungi secrete lignin‐degrading enzymes (e.g., laccase and polyphenol oxidases) along with cellulose‐degrading enzymes (e.g., cellulase), decomposing lignocellulose to provide nutrients for mycelial growth [34]. Specifically, laccase‐type polyphenol oxidases catalyze phenolic oxidation in lignin to form quinones. These quinones subsequently participate in nonenzymatic reactions that induce structural modifications, facilitating lignin degradation. Furthermore, polyphenol oxidases act synergistically with other ligninolytic enzymes (e.g., laccase) to enhance decomposition efficiency [35].

The primordial differentiation phase (Day 60) represents a critical transition period from vegetative to reproductive growth in *A. polytricha* [36]. During early mycelial colonization (Day 25), activities of cellulase, laccase, and polyphenol oxidase peaked, followed by a significant decline by Day 60. This temporal pattern aligns with extracellular enzyme dynamics documented in the vegetative growth stages of related basidiomycetes *Lentinula edodes* and *A. auricula* [21, 22, 37]. The observed activity reduction likely results from substrate depletion: In initial growth phases, abundant lignocellulose resources drive efficient enzymatic decomposition to support biomass accumulation; postpeak substrate consumption triggers the downregulation of extracellular enzyme synthesis.

Substrate composition significantly modulates extracellular enzyme profiles in *A. polytricha* mycelia [30]. At the mycelial colonization stage (Day 25), cellulase activity peaked at 20.9 U/g under 0% cotton straw:50% coix seed straw:50% wheat straw (Figure 2f), while polyphenol oxidase reached 891.1 U/g in pure wheat straw substrate (0:0:100; Figure 2j). These substrate‐specific activity patterns demonstrate differential lignocellulose degradative capacities. At the postripening phase (Day 40), characterized by completed primary degradation and nutrient reservoir formation, laccase activity maximized (143.5 U/g) under 66.7% cotton straw:16.7% coix seed straw:16.7% wheat straw (Figure 2c). Cellulase peaked at 19.1 U/g in 50% cotton straw:50% coix seed straw:0% wheat straw (Figure 2g), indicating wheat straw′s negligible impact on extracellular enzymes during this phase. At the primordial differentiation phase (Day 60, critical for fructification), polyphenol oxidase dominated extracellular enzyme activity, reaching 400.6 U/g under equal‐part substrate (33.3% each straw type; Figure 2l). In summary, these results demonstrate that substrate composition affects the degradation capacity of different extracellular enzymes at various time points and plays a coordinated role in the extracellular enzyme system.

#### 3.1.4. Influence of Agricultural Waste as Substrate on Agronomic Traits of *A. polytricha*

Substrate composition critically influences the growth cycle and yield of *A. polytricha*, as established by Akbar et al. [18]. Table 3 details substrate effects on agronomic traits: The control group (CK) reached harvest maturity at 96.47 ± 0.21 days, whereas treatment T6 required 97.10 ± 0.27 days. Although CK exhibited a higher yield per bag (122.5 ± 2.43 g) compared to T6 (118.2 ± 1.35 g), T6 achieved significantly greater biological efficiency. This enhanced efficiency in T6 correlates with its substrate formulation containing wood shavings.

**Table 3:** Average mycelial growth rate, yield, biological efficiency, soaking rate, and productivity obtained for *A. polytricha* in different proportions of the substrate.

**Substrate**	**Harvest time/day**	**Total yield (g/bag)**	**Biologic efficiency %**	**Soaking rate %**
CK	96.47 ± 0.21^i^	122.5 ± 2.43^a^	46.83^b^	10.16 ± 0.52^e^
T1	102.34 ± 0.14^d^	66.97 ± 1.59^f^	32.26^f^	12.57 ± 0.89^ab^
T2	100.58 ± 0.29^e^	73.07 ± 3.41^e^	37.20^d^	12.31 ± 0.43^ab^
T3	103.55 ± 0.13^c^	80.77 ± 0.84^d^	37.88^d^	12.38 ± 0.38^ab^
T4	103.31 ± 0.06^c^	43.09 ± 2.71^g^	16.40^g^	10.99 ± 0.78^cd^
T5	98.45 ± 0.33^f^	73.5 ± 1.15^e^	35.40^e^	12.76 ± 0.61^a^
T6	97.1 ± 0.27^h^	118.2 ± 1.35^b^	54.62^a^	11.46 ± 0.4^abc^
T7	97.54 ± 0.27^g^	94.67 ± 1.71^c^	40.32^c^	10.11 ± 1.23^de^
T8	104.39 ± 0.33^b^	15.47 ± 1.22^i^	6.18^i^	11.83 ± 0.78^abc^
T9	103.52 ± 0.37^c^	22.13 ± 0.99^h^	9.66^h^	11.32 ± 0.92^bcd^
T10	105.4 ± 0.11^a^	16.47 ± 1.01^i^	7.39^i^	9.65 ± 0.63^f^

*Note:* Values are expressed as means ± standard errors of means, *n* = 3. Lack of letters in common indicates statistically significant differences (Duncan′s test, *p* < 0.05) for comparison of the treatments between the different substrates (lowercase letters).

All treatments except T7 and T9 showed reduced contamination rates compared to CK. Contamination rate serves as a key quality indicator for *A. auricula* cultivation [38]. Crucially, substrate C/N ratio profoundly impacts yield and biological efficiency, with elevated ratios exerting negative effects [39]. As quantified in Table 1, experimental substrates exhibited C/N ratios ranging from 31:1 to 55:1—all supporting mycelial growth. Optimal mycelial development and yield occur at C/N 60:1 [40], whereas ratios below 23.7:1 inhibit *L. crinitus* myceliation and completely block fructification [41]. Notably, T6 (C/N 49:1) achieved high yield and efficiency, contrasting with T9 (C/N 50:1), which underperformed, indicating multifactorial yield regulation beyond the C/N ratio alone.

**Figure 3:**
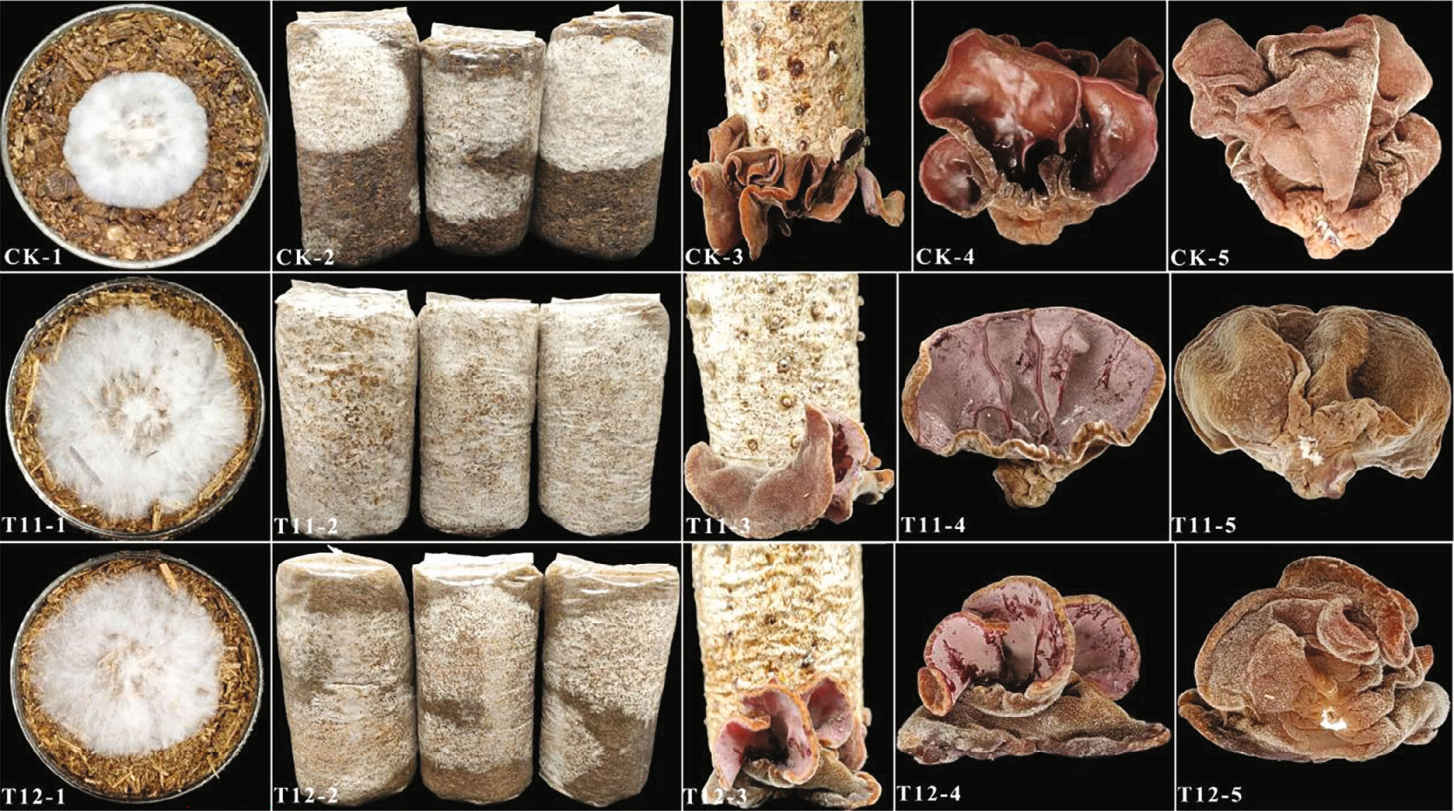
Effects of optimized straw formula on cultivation of *A. polytricha.* CK‐1, T11‐1, and T12‐1: mycelial growth status; CK‐2, T11‐2, and T12‐2: the growth of bacterial package on the 20th day; CK‐3, T11‐3, and T12‐3: harvesting diagram of subentity; CK‐4, T11‐4, and T12‐4: *A. polytricha* on the fresh front; CK‐5, T11‐5, and T12‐5: fresh back.

Mixture experimental design represents a validated methodology for optimizing edible mushroom substrate formulations, enabling identification of substrate ratios that maximize mycelial growth efficiency [42]. *A. polytricha* cultivated with straw‐based optimized substrates (Figure 3) demonstrated superior morphological characteristics: Fruiting bodies from treatments T11 and T12 exhibited enhanced flesh density and brighter coloration compared to the control. This indicates that substrate composition significantly influences *A. polytricha* fructification under standardized cultivation conditions. As quantified in Table 4, T11 achieved the shortest production cycle (70.33 ± 0.40 days) and the highest yield (89.2 ± 2.32 g/bag), followed by T12 (76.70 ± 0.56 days; 68.03 ± 2.50 g/bag). These optimized formulations were cultivated during autumn–winter seasons in outdoor bag systems. In contrast, the control group showed extended growth cycles and reduced yields. These findings demonstrate that agricultural waste substrates with optimized C/N ratios and mixture designs critically regulate *A. polytricha* productivity. While wood shaving formulation (T6) balanced yield and biological efficiency, the straw‐optimized substrates (T11–T12) produced superior quality mushrooms with 10–16 days shorter cultivation cycles relative to conventional substrates.

**Table 4:** Optimize the time of full bag, harvest time, and monaural weight of *A. polytricha*.

**Substrate**	**CK**	**T11**	**T12**
Harvest time/day	86.77 ± 0.31^a^	70.33 ± 0.4^b^	76.7 ± 0.56^b^
Total yield (g)	58.63 ± 1.82^c^	89.2 ± 2.32^a^	68.03 ± 2.5^b^
Biologic efficiency %	23.45^c^	35.68^a^	27.21^b^
Productivity (g/day)	0.676^c^	1.268^a^	0.887^b^
Soaking rate %	9.07 ± 0.88^b^	8.45 ± 0.42^c^	9.36 ± 0.44^a^

*Note:* The results were expressed as mean ± SD value (*n* = 3). Different letters mean significant differences (*p* < 0.05).

### 3.2. Influence of Substrate Matrix Composition on Fruiting Bodies, Nutrients, and Mineral Nutrition

Edible mushrooms are valued as nutrient‐dense sources of nonstarchy carbohydrates, dietary fiber, proteins with complete amino acid profiles, essential minerals, and vitamins, serving as functional meat alternatives in plant‐based diets [43]. Prior research confirms substrate‐dependent nutrient variation in *A. cornea* [44]. This study is aimed at cultivating *A. polytricha* using agroresidues and evaluating its nutritional profiles across substrate formulations. Nutritional composition analysis (Table 5) revealed significantly higher crude protein in T7 (24.84*%* ± 0.06*%* DW) versus control and other formulations (*p* < 0.05). Similarly, T1 exhibited elevated total sugars (17.79*%* ± 0.21*%* DW). While substrate minimally affected crude fat, Ca, Mg, and Cu contents, it substantially altered crude protein, total sugars, Fe, and Zn concentrations. These findings align with Ye et al. [45], who documented 73.4% crude protein increase (14.85% → 25.74%), 284.9% Fe elevation (25.64 → 98.58 mg/kg), and 892.2% Se enhancement (5.13 → 50.90 *μ*g/kg) in *A. cornea* cultivated on *Tetradium ruticarpum* sawdust versus mixed hardwood chips.

**Table 5:** Nutritional quality of fruiting bodies of *A. polytricha* in different cultivation formulas.

**Formulation**	**Fat/%**	**Protein/%**	**Total sugar/%**	**Mineral element**					
				**Ca/%**	**Mg/%**	**Fe (mg/kg)**	**Mn (mg/kg)**	**Cu (mg/kg)**	**Zn (mg/kg)**
CK	5.55 ± 0.2^f^	9.42 ± 0.08^h^	7.95 ± 0.08^m^	0.4557^ab^	0.2137^b^	28.12 ± 0.12^k^	29.46 ± 0.19^a^	3.47 ± 0.2^f^	10.56 ± 0.2^k^
T1	7.37 ± 0.08^c^	18.52 ± 0.23^b^	17.79 ± 0.21^a^	0.3977^cd^	0.246^a^	122 ± 0.01^b^	16.51 ± 0.28^e^	4.46 ± 0.17^a^	77.61 ± 0.3^a^
T2	8.28 ± 0.09^b^	14.24 ± 0.13^f^	16.06 ± 0.19^b^	0.4533^ab^	0.2137^b^	127.4 ± 0^a^	15.1 ± 0.11^f^	3.85 ± 0.15^cd^	63.45 ± 0.12^b^
T3	6.53 ± 0.16^e^	16.18 ± 0.2^c^	10.34 ± 0.12^h^	0.3547^de^	0.1857^f^	79.3 ± 0.05^e^	14.43 ± 0.39^g^	4.1 ± 0.06^b^	39.66 ± 0.51^e^
T4	7.51 ± 0.20^c^	14.5 ± 0.16^ef^	12.46 ± 0.03^e^	0.3363^ef^	0.1957^e^	60.04 ± 0^h^	18.86 ± 0.08^d^	3.98 ± 0.05^bc^	38.27 ± 0.19^g^
T5	5.76 ± 0.11^f^	15.56 ± 0.11^d^	15.75 ± 0.08^c^	0.3123^efg^	0.201^d^	74.22 ± 0.06^g^	15.46 ± 0.25^f^	3.66 ± 0.02^def^	52.73 ± 0.05^d^
T6	4.95 ± 0.17^g^	14.83 ± 0.05^e^	9.36 ± 0.04^j^	0.26^gh^	0.212^b^	75.96 ± 0.11^f^	11.49 ± 0.29^k^	3.06 ± 0.03^g^	62.76 ± 0.25^c^
T7	7.55 ± 0.17^c^	24.84 ± 0.06^a^	9.84 ± 0.06^i^	0.2977^fg^	0.1853^f^	23.19 ± 0.02^m^	13.25 ± 0.37^i^	3.14 ± 0.01^g^	37.22 ± 0.09^h^
T8	6.52 ± 0.23^e^	15.72 ± 0.06^d^	12.79 ± 0.10^d^	0.4237^bc^	0.167^g^	39.8 ± 0.29^j^	22.16 ± 0.18^c^	3.6 ± 0.06^ef^	23.44 ± 0.08^i^
T9	7.66 ± 0.23^c^	15.65 ± 0.32^d^	11.44 ± 0.02^g^	0.4933^a^	0.2073^c^	106.28 ± 0.16^c^	24.94 ± 0.1^b^	3.76 ± 0.1^cde^	38.69 ± 0.21^f^
T10	6.68 ± 0.22^e^	16.25 ± 0.76^c^	11.84 ± 0.13^f^	0.2207^h^	0.162^h^	96.86 ± 2.35^d^	13.7 ± 0.07^h^	3.84 ± 0.29^cd^	18.56 ± 0.26^j^
T11	9.09 ± 0.18^a^	11.54 ± 0.11^g^	8.41 ± 0.18^l^	0.328e^f^	0.1607^h^	26.43 ± 0.07^l^	9.56 ± 0.28^l^	3.02 ± 0.06^g^	10.5 ± 0.25^k^
T12	6.98 ± 0.08^d^	7.43 ± 0.04^i^	8.69 ± 0.09^k^	0.2623g^h^	0.1613^h^	47.95 ± 0.48^i^	12.16 ± 0.1^j^	2.72 ± 0.03^h^	9.43 ± 0.14^l^

*Note:* The results were expressed as mean ± SD value (*n* = 3). Different letters mean significant differences (*p* < 0.05).

Edible mushrooms bioaccumulate essential minerals, including Fe, Mn, Cu, and Zn, during growth [46], with significant compositional differences between cultivated and wild specimens [47]. As quantified in Table 5, straw‐based formulations significantly enhanced Fe and Zn concentrations in *A. polytricha*. Specifically, T2 exhibited elevated Fe (127.4 mg/kg DW) and Zn (77.61 ± 0.3 mg/kg DW) versus control (*p* < 0.05), while Mn was significantly reduced. No significant differences occurred in Ca, Mg, or Cu concentrations.

Xu et al. [48] reported that cultivating *A. auricula* using corn straw could increase the content of ash, protein, Cu, and Fe in its substrate while decreasing the content of Zn, Mg, Mn, and colloidal substances. Straw resources contain significant macronutrient concentrations, notably nitrogen (0.65%–1.82% *w*/*w*), P (0.08%–0.196% *w*/*w*), and K (1.02%–1.94% *w*/*w*) on a dry weight basis [9, 12]. Consequently, we hypothesize that valorizing agricultural residues as cultivation substrates may yield nutrient‐enriched edible mushrooms. Nevertheless, mechanistic linkages between straw nutrient profiles and resultant fungal nutritional composition remain poorly characterized, warranting systematic investigation.

### 3.3. Influence of Agricultural Waste as Substrate on the Antioxidant Properties of *A. polytricha*

Free radical scavenging represents a primary mechanism through which antioxidants inhibit lipid oxidation. This implies that evaluating edible mushroom antioxidants via a single assay system may inadequately reflect their biological significance. Consequently, a comprehensive assessment of antioxidant efficacy requires complementary assay systems (e.g., DPPH, ABTS, and ORAC) to capture multifunctional activities. Critically, cultivation substrates significantly modulate mushroom antioxidant properties [49]. We evaluated the antioxidant capacity of *A. polytricha* fruiting bodies cultivated under different substrate formulations.

Figure 4a quantifies DPPH radical scavenging capacity of *A. polytricha* fruiting bodies across treatments, benchmarked against ascorbic acid (VC; 0.5–2.5 mg/mL). Substrate‐dependent variations in scavenging activity were observed. Concentration–response analysis confirmed enhanced scavenging with increasing extract concentration [50], with T1 exhibiting maximal activity (57.78%) at 2.5 mg/mL. Notably, straw‐cultivated specimens demonstrated 32.7% higher scavenging capacity versus wood chip–cultivated controls, attributable to substrate‐derived bioactive compound differentials. •OH, among the most reactive oxygen species (ROS), induce oxidative damage to DNA, proteins, and biomacromolecules [51]. Figure 4b compares the hydrogen peroxide scavenging capacities of *A. polytricha* fruiting bodies from various substrates against ascorbic acid (positive control; 0.5–2.5 mg/mL). Paradoxically, •OH scavenging capacity decreased with increasing concentration, exemplified by T3 declining from 28.64*%* ± 1.2% to 18.07*%* ± 0.8*%*—contradicting established concentration–response relationships [3]. Figure 4c demonstrates ferric reduction activity (FRAP assay) of *A. polytricha* extracts [52]. Reducing power increased dose‐dependently, though wood chip–cultivated specimens exhibited 31.2% lower activity versus straw‐grown counterparts (e.g., T6: 0.19 → 0.26 vs. sawdust: 0.17 → 0.19 at 5 mg/mL).

Figure 4Antioxidant capacity of *A. polytricha* in vitro. (a) DPPH radical scavenging activity. (b) Hydroxyl radical scavenging activity. (c) Ferric reducing power.

**Figure:**
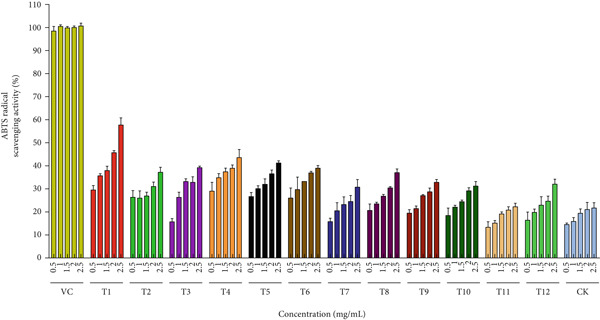
(a)

**Figure:**
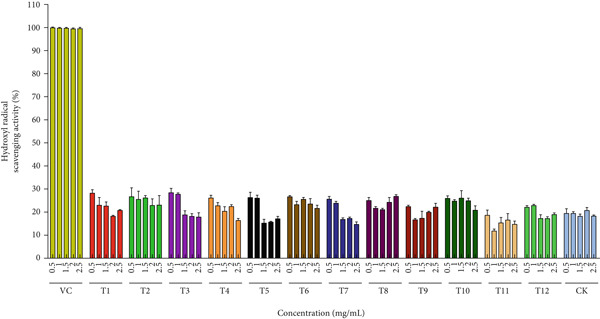
(b)

**Figure:**
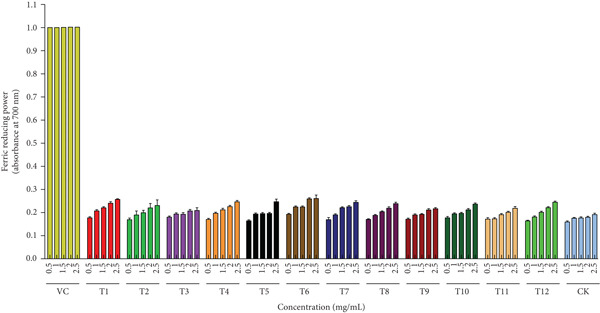
(c)

Integrating DPPH/•OH scavenging and FRAP data, straw‐cultivated *A. polytricha* showed 40.3% higher aggregate antioxidant capacity than sawdust‐cultivated specimens yet remained 22.7%–58.1% below ascorbic acid controls. These variations correlate with substrate‐modulated bioactive compounds (flavonoids, phenolics, and *β*‐glucans) [53].

## 4. Conclusion

This study successfully demonstrates the potential of agricultural waste as a substrate for cultivating the nutritionally valuable edible mushroom *A. polytricha*. Cotton straw, coix seed straw, and wheat straw substrates significantly enhanced mycelial growth rate and extracellular enzymatic activity in *A. polytricha*, confirming their viability as cultivation media. Crucially, substrate combinations outperformed individual applications: Formulation T6 achieved a biological efficiency of 54.62%, while T11 shortened the growth cycle by 16.44 days. *A. polytricha* cultivated on straw‐based substrates exhibited elevated levels of total sugars, fats, crude protein, and trace elements (Fe and Zn). Furthermore, DPPH radical scavenging capacity was enhanced. The “Straw for Wood” method provides an environmentally sustainable and cost‐effective cultivation strategy, highlighting the untapped potential of agricultural waste. A limitation is the absence of long‐term data on substrate effects on mushroom safety and nutritional quality, warranting further investigation.

## Disclosure

All authors have read and agreed to the published version of the manuscript.

## Conflicts of Interest

The authors have received a research grant from the Guizhou Provincial Support Fund of Science and the Technology and National Natural Science Foundation of China.

## Author Contributions

Zaili Qin: methodology, formal analysis, and writing—original draft. Nan Wu: resources. Shihui Mei: software. Jiangtao Xie: data curation. Entaj Tarafder: visualization and revising the manuscript. Changtian Li: visualization and revising the manuscript. Fenghua Tian: revising manuscript, project administration, and funding acquisition.

## Funding

The study was funded by the Guizhou Provincial Support Fund of Science and Technology, QKH [2021] General 199; the National Natural Science Foundation of China, 10.13039/501100001809, NSFC: 32260044; the GZMARSEdible Fungi, GZMARS‐SYJ‐2021‐2025; and the Tianjin Synthetic Biotechnology Innovation Capacity Improvement Project, TSBICIP‐CXRC‐006.

## Data Availability

Data will be made available on request.
